# Supplementary material for: Amino Derivatives of Diaryl Pyrimidines and Azolopyrimidines as Protective Agents against LPS-Induced Acute Lung Injury
Source: Molecules. 2023 Jan 11;28(2):741. doi: 10.3390/molecules28020741 (PMC9863002; doi:10.3390/molecules28020741)
Supplement: Supplementary file 1 [file molecules-28-00741-s001.zip › molecules-2113133-supplementary.pdf]

Materials and Methods

^1H NMR spectra were recorded in DMSO-d_6 solution on device "Bruker DRX-400" using TMS and DMSO-d_6 as an internal standard. The following abbreviations are used for a multiplicity of NMR signals: s – singlet, d – doublet, t – triplet, q – quartet, dd – double doublet, m – multiplet, br – broaded. Electrospray ionization mass spectra were recorded for positive ions on a qTOF maXis Impact HD ultra-high resolution mass spectrometer (Bruker Daltonics, USA) with a standard ionization source in the mass range 50-2500 Da by injection analysis for sample solutions in acetonitrile using a syringe pump inlet (model No. 601553 kdScientific inc., USA); solution infusion rate of 240 μL/h) in the modified preset method "Direct_Infusion 100-1000". Calibration of the mass scale - external, according to the signals of a solution of lithium acetate by HPC methods or improved quadratic. All data were collected and processed using the Compass for oTof series 1.7 software package (oTOF Control 3.4; Bruker Compass DataAnalysis 4.2). IR spectra were recorded on a Spectrum One FT-IT spectrometer (Perkin Elmer) in a range of 4000—400 cm–1 using a diffuse reflectance attachment. Elemental analysis was performed on a PerkinElmer PE 2400 elemental analyzer. Melting points were determined on a Stuart SMP3 and are uncorrected. The monitoring of the reaction progress was performed by using TLC on Silufol UV254 plates. Column chromatography was performed on Chromagel (silica gel, 400 mesh).

Synthesis

General procedure for the preparation of nitro- and dinitrochalcones 3 (i, Scheme 2.1*):*

The nitrochalcones **3a–c** were obtained by a known method [15]. The dinitrochalcones **3d,e** were obtained as follows. To a solution of 2 g (0.012 mol) of nitroacetophenone and 2.4 g (0.015 mol) of nitrobenzaldehyde in 30 mL of glacial acetic acid, H_3BO_3 (0.7 g, 0.012 mol) was added and the mixture was refluxed for 12 h. The solution was cooled to 25 °C. The formed precipitate was filtered and washed on the filter with 5 portions (10 mL) of an aqueous solution of acetic acid (1:1) and finally with water. The product was dried and crystallized from ethyl acetate. The physical characteristics of chalcones corresponded to the literature data [19].

General procedure for the preparation of nitro derivatives of pyrimidines and triazolo[1,5-a]pyrimidines (ii, Scheme 2.1*)*

To a solution chalcone **3** (1.6 mmol) and acetamidine hydrochloride, or guanidine carbonate, or 2-methylisothiuronium iodide) (2 mmol) in 20 mL of dimethylformamide, K_2CO_3 (3 mmol) was added. The mixture was stirred at 100 °C for 32 h. Water was added to the mixture upon completion of the process, and the formed precipitate was filtered. The product, eluting with chloroform or chloroform-THF (9:1) in the case of triazoloprimidines, was purified chromatographically on a preparative column (SiO_2). The compound 4,6-bis(4-nitrophenyl)pyrimidin-2-amine was crystallized from DMF.

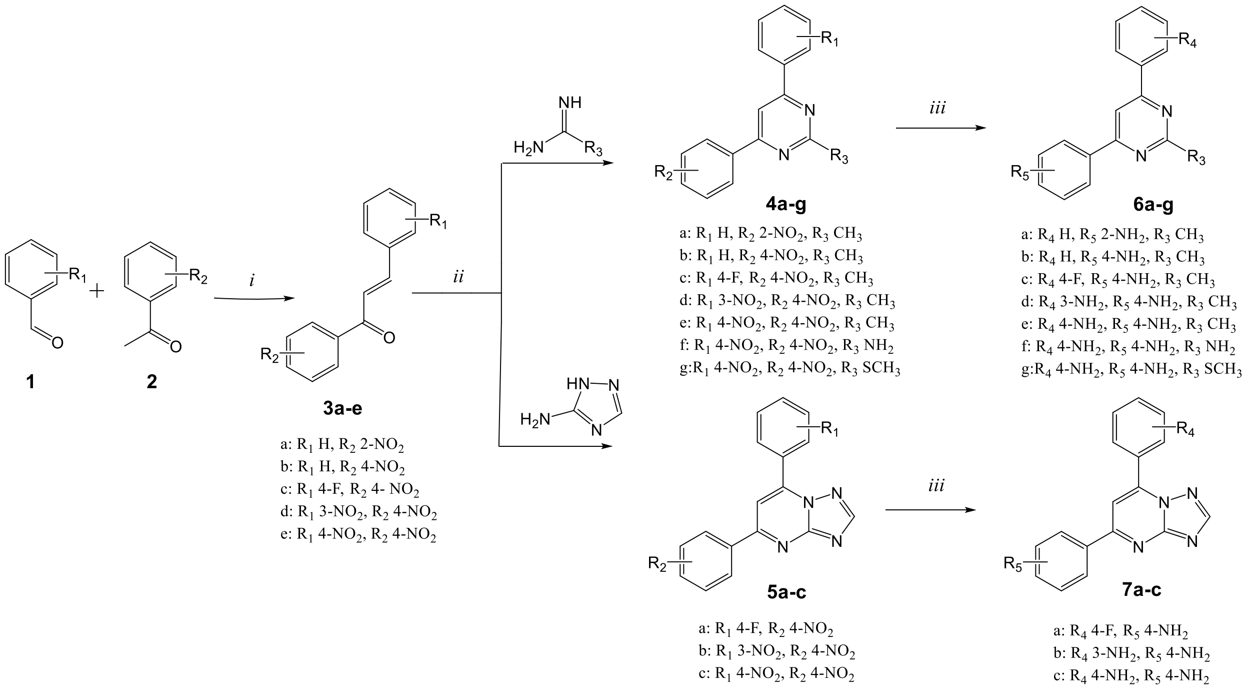

Scheme S2.1

2-Methyl-4-(2-nitrophenyl)-6-phenylpyrimidine ***4a***

Light yellow solid; mp = 141-143 °C. Yield: 590 mg (68%). ^1H NMR (400 MHz, DMSO-d$_6$) δ, 8.31 (m, 2 H, Ar), 8.26 (s, 1 H, CH, pyrimidine), 8.07 (dd, 1 H, *J* = 0.9, 8.0 Hz, Ar), 7.97 (dd, 1 H, *J* = 1.2, 7.6 Hz, Ar), 7.88 (td, 1 H, *J* = 0.9, 7.1, 8.0 Hz, Ar), 7.78 (td, 1 H, *J* = 1.2, 7.1, 7.6 Hz, Ar), 7.55 – 7.58 (m, 3 H, Ar), 2.66 (s, 3 H, Me). ^{13}C NMR (101 MHz, DMSO-d$_6$) δ 167.12, 164.07, 163.45, 148.76, 136.01, 132.99, 132.38, 131.43, 131.22, 130.93, 128.92 (2 C), 127.24 (2 C), 124.44, 112.37, 25.71. HRMS(ESI): calculated for $C_{17}H_{13}NaN_3O_2$ $[M + Na]^+$: 314.0900, found: 314.0901; IR ν = 3063, 3037, 2969, 2878, 1573, 1526, 1441, 1367, 1304, 1242, 1155, 1106, 880, 857, 823, 781, 746, 682 cm^{-1}. Anal. Calcd for $C_{17}H_{13}N_3O_2$: C, 70.09; H, 4.50; N, 14.42. Found: C, 70.03; H, 4.56; N, 14.40.

2-Methyl-4-(4-nitrophenyl)-6-phenylpyrimidine ***4b***

Light yellow solid; mp = 197-199 °C. Yield: 605 mg (70%). ^1H NMR (400 MHz, DMSO-d$_6$) δ, 8.61 (m (AA′BB′), 2 H, Ar), 8.57 (s, 1 H, CH, pyrimidine), 8.40 (m (AA′BB′), 2 H, Ar), 8.37 (m, 2 H, Ar), 7.58 – 7.61 (m, 3 H, Ar), 2.79 (s, 3 H, Me). ^{13}C NMR (101 MHz, DMSO-d$_6$) δ 167.85, 164.51, 161.65, 148.81, 142.50, 136.17, 131.19, 128.86 (2 C), 128.57 (2 C), 127.33 (2 C), 123.87 (2 C), 110.69, 26.04. HRMS(ESI): calculated for $C_{17}H_{14}N_3O_2$ $[M + H]^+$: 292.1081, found: 292.1080; IR ν = 3107, 3073, 3054, 1576, 1535, 1511, 1441, 1415, 1397, 1344, 1233, 1105, 1012, 855, 819, 743, 687, 641 cm^{-1}. Anal. Calcd for $C_{17}H_{13}N_3O_2$: C, 70.09; H, 4.50; N, 14.42. Found: C, 70.05; H, 4.58; N, 14.39.

4-(4-Fluorophenyl)-2-methyl-6-(4-nitrophenyl)pyrimidine ***4c***

Light yellow solid; mp = 280-283 °C. Yield: 676 mg (73%). ^1H NMR (400 MHz, DMSO-d$_6$) δ, 8.62 (m (AA′BB′), 2 H, Ar), 8.60 (s, 1 H, CH, pyrimidine), 8.46 (m (AA′BB′C), 2 H, Ar), 8.41 (m (AA′BB′), 2 H, Ar), 7.43 (m (AA′BB′C), 2 H, Ar), 2.79 (s, 3 H, Me). ^{13}C NMR (101 MHz, DMSO-d$_6$) δ 167.83, 165.06, 163.24 (d, 1 C, *J* = 106.9 Hz), 161.72, 148.85, 142.45, 132.66 (d, 1 C, *J* = 7.02 Hz), 129.83 (d, 2 C, *J* = 23.04 Hz), 128.57 (2 C), 123.86 (2 C), 115.82 (d, 2 C, *J* = 56.3 Hz), 110.53, 26.01. HRMS(ESI): calculated for $C_{17}H_{13}FN_3O_2$ $[M + H]^+$: 310.0992, found: 310.0986; IR ν = 3126, 3048, 2925, 2846, 1600, 1577, 1541, 1509, 1418, 1346, 1228, 1165, 1102, 1012, 839, 754 cm^{-1}. Anal. Calcd for $C_{17}H_{12}FN_3O_2$: C, 66.02; H, 3.91; N, 13.59. Found: C, 66.05; H, 3.90; N, 13.61.

2-Methyl-4-(3-nitrophenyl)-6-(4-nitrophenyl)pyrimidine ***4d***

Light yellow solid; mp = 209-211 °C. Yield: 725 mg (72%). ^1H NMR (400 MHz, DMSO-d$_6$) δ, 9.12 (d, 1 H, *J* = 1.8 Hz, Ar), 8.81 (d, 1 H, *J* = 7.9 Hz, Ar), 8.73 (s, 1 H, CH, pyrimidine), 8.62 (m (AA′BB′), 2 H, Ar), 8.42 (dd, 1 H, *J* = 1.8, 8.1 Hz, Ar), 8.39 (m (AA′BB′), 2 H, Ar), 7.88 (t, 1 H, *J* = 8.0 Hz, Ar), 2.82 (s, 3 H, Me). ^{13}C NMR (101 MHz, DMSO-d$_6$) δ 168.07, 162.18, 162.11, 148.93, 148.45, 142.09, 137.82, 133.63, 130.54, 128.69 (2 C), 125.64, 123.87 (2 C), 121.73, 111.22, 26.02. HRMS(ESI): calculated for $C_{17}H_{12}KN_4O_4$ $[M + K]^+$:

375.0490, found: 375.0487; IR ν = 3109, 3088, 2967, 2925, 2854, 1574, 1538, 1516, 1416, 1392, 1348, 1244, 1106, 854, 811, 752, 735, 714, 677 cm^{-1}. Anal. Calcd for $C_{17}H_{12}N_4O_4$: C, 60.71; H, 3.60; N, 16.66. Found: C, 60.73; H, 3.59; N, 16.67.

2-Methyl-4,6-bis(4-nitrophenyl)pyrimidine ***4e***

Light yellow solid; mp = 260-263 °C. Yield: 712 mg (71%). ^{1}H NMR (400 MHz, DMSO-d$_6$) δ, 8.73 (s, 1 H, CH, pyrimidine), 8.63 (m (AA′BB′), 4 H, Ar), 8.42 (m (AA′BB′), 4 H, Ar), 2.83 (s, 3 H, Me). ^{13}C NMR (101 MHz, DMSO-d$_6$) δ 168.18, 162.31 (2 C), 149.01 (2 C), 142.13 (2 C), 128.73 (4 C), 123.98 (4 C), 111.89, 26.05. HRMS(ESI): calculated for $C_{17}H_{13}N_4O_4$ [M + H]$^+$: 337.0931, found: 337.0930; IR ν = 3114, 3073, 2851, 1575, 1543, 1525, 1508, 1414, 1345, 1317, 1100, 850, 815, 744 cm^{-1}. Anal. Calcd for $C_{17}H_{12}N_4O_4$: C, 60.71; H, 3.60; N, 16.66. Found: C, 60.69; H, 3.62; N, 16.65.

4,6-bis(4-Nitrophenyl)pyrimidin-2-amine ***4f***

Light yellow solid; mp = 323-326 °C. Yield: 710 mg (71%). ^{1}H NMR (400 MHz, DMSO-d$_6$) δ, 8.51 (m (AA′BB′), 4 H, Ar), 8.39 (m (AA′BB′), 4 H, Ar), 8.01 (s, 1 H, CH, pyrimidine), 7.12 (s, 2 H, NH_2). ^{13}C NMR (101 MHz, DMSO-d$_6$) δ 164.06, 163.26 (2 C), 148.69 (2 C), 143.02 (2 C), 128.34 (4 C), 123.80 (4 C), 103.62. HRMS(ESI): calculated for $C_{16}H_{11}KN_5O_4$ [M + K]$^+$: 376.0443, found: 376.0440; IR ν = 3483, 3342, 3221, 3128, 3080, 1639, 1566, 1553, 1523, 1509, 1467, 1349, 1220, 1106, 1012, 873, 855, 836, 819, 756, 706 cm^{-1}. Anal. Calcd for $C_{16}H_{11}N_5O_4$: C, 56.98; H, 3.29; N, 20.76. Found: C, 56.95; H, 3.31; N, 20.75.

2-(Methylthio)-4,6-bis(4-nitrophenyl)pyrimidine ***4g***

Light yellow solid; mp = 273-276 °C. Yield: 695 mg (69%). ^{1}H NMR (400 MHz, DMSO-d$_6$) δ, 8.62 (m (AA′BB′), 4 H, Ar), 8.61 (s, 1 H, CH, pyrimidine), 8.41 (m (AA′BB′), 4 H, Ar), 2.71 (s, 3 H, Me). ^{13}C NMR (101 MHz, DMSO-d$_6$) δ 172.44, 162.49 (2 C), 149.17 (2 C), 141.59 (2 C), 128.84 (4 C), 123.99 (4 C), 110.20, 13.79. HRMS(ESI): calculated for $C_{18}H_{13}N_4O_6S$ [M + CHOO]$^-$: 413.0561, found: 413.0561; IR ν = 3113, 3082, 2930, 2843, 1672, 1564, 1531, 1406, 1346, 1325, 1305, 1251, 1115, 1091, 1074, 1010, 847, 815, 746, 690 cm^{-1}. Anal. Calcd for $C_{17}H_{12}N_4O_4S$: C, 55.43; H, 3.28; N, 15.21. Found: C, 55.49; H, 3.26; N, 15.24.

7-(4-Fluorophenyl)-5-(4-nitrophenyl)-[1,2,4]triazolo[1,5-a]pyrimidine ***5a***

Light yellow solid; mp = 244-246 °C. Yield: 535 mg (53%). ^{1}H NMR (400 MHz, DMSO-d$_6$) δ, 8.81 (s, 1 H, CH, triazolopyrimidine), 8.68 (m (AA′BB′), 2 H, Ar), 8.21 – 8.45 (m (AA′BB′, AA′BB′C), 4 H, Ar), 8.34 (s, 1 H, CH, triazolopyrimidine), 7.55 (m (AA′BB′C), 2 H, Ar). ^{13}C NMR (101 MHz, DMSO-d$_6$) δ 164.95, 162.96, 158.27, 156.08 (d, 1 C, *J* = 130.9 Hz), 148.97, 146.99, 141.95, 132.66 (d, 2 C, *J* = 11.7 Hz), 129.18 (2 C), 126.11 (d, 1 C, *J* = 4.02 Hz), 123.98 (2 C), 115.69 (d, 2 C, *J* = 28.6 Hz), 107.34. HRMS(ESI): calculated for $C_{17}H_{11}FN_5O_2$ [M + H]$^+$: 336.0891, found: 336.0895; IR ν = 3110, 3073, 1717, 1606, 1546, 1532,

1513, 1347, 1302, 1283, 1260, 1230, 1190, 1168, 1143, 1109, 836, 760 cm^{-1}. Anal. Calcd for $C_{17}H_{10}FN_5O_2$: C, 60.90; H, 3.01; N, 20.89. Found: C, 60.93; H, 2.99; N, 20.91.

7-(3-Nitrophenyl)-5-(4-nitrophenyl)-[1,2,4]triazolo[1,5-a]pyrimidine ***5b***

Light yellow solid; mp = 258-260 °C. Yield: 520 mg (48%). ^{1}H NMR (400 MHz, DMSO-d$_6$) δ, 9.21 (t, 1 H, *J* = 1.8 Hz, Ar), 8.86 (s, 1 H, CH, triazolopyrimidine), 8.74 (dkt, 1 H, *J* = 0.6, 1.6, 7.8 Hz, Ar), 8.71 (m (AA′BB′), 2 H, Ar), 8.54 (dkt, 1 H, *J* = 1.4, 2.3, 8.3 Hz, Ar), 8.52 (s, 1 H, CH, triazolopyrimidine), 8.45 (m (AA′BB′), 2 H, Ar), 7.99 (t, 1 H, *J* = 8.1 Hz, Ar). ^{13}C NMR (101 MHz, DMSO-d$_6$) δ 158.43, 156.63, 155.60, 149.06, 147.68, 145.70, 141.76, 136.25, 131.11, 130.26, 129.25 (2 C), 126.26, 124.08, 124.07 (2 C), 108.19. HRMS(ESI): calculated for $C_{17}H_{11}N_6O_4$ [M + H]$^+$: 363.0847, found: 363.0842; IR *ν* = 3125, 3106, 3075, 3053, 1608, 1530, 1517, 1342, 1294, 1261, 1240, 1180, 1101, 932, 852, 814, 760, 738, 726, 673 cm^{-1}. Anal. Calcd for $C_{17}H_{10}N_6O_4$: C, 56.36; H, 2.78; N, 23.20. Found: C, 56.39; H, 2.75; N, 23.21.

5,7-bis(4-Nitrophenyl)-[1,2,4]triazolo[1,5-a]pyrimidine ***5c***

Light yellow solid; mp = 286-288 °C. Yield: 456 mg (42%). ^{1}H NMR (400 MHz, DMSO-d$_6$) δ, 8.84 (s, 1 H, CH, triazolopyrimidine), 8.68 (m (AA′BB′), 2 H, Ar), 8.57 (m (AA′BB′), 2 H, Ar), 8.51 (m (AA′BB′), 2 H, Ar), 8.46 (s, 1 H, CH, triazolopyrimidine), 8.44 (m (AA′BB′), 2 H, Ar). ^{13}C NMR (101 MHz, DMSO-d$_6$) δ 158.44, 156.64, 155.60, 149.06, 149.01, 145.89, 141.73, 135.61, 131.46 (2 C), 129.24 (2 C), 124.08 (2 C), 123.51 (2 C), 108.47. HRMS(ESI): calculated for $C_{17}H_{11}N_6O_4$ [M + H]$^+$: 363.0840, found: 363.0847; IR *ν* = 3109, 3068, 3050, 2918, 2849, 1614, 1598, 1546, 1519, 1488, 1347, 1305, 1295, 1259, 1235, 1182, 1139, 1107, 1003, 848, 836, 753, 696 cm^{-1}. Anal. Calcd for $C_{17}H_{10}N_6O_4$: C, 56.36; H, 2.78; N, 23.20. Found: C, 56.33; H, 2.80; N, 23.19.

General procedure for the preparation of aniline-substituted pyrimidines and triazolo[1,5-a]pyrimidines (iii, Scheme 2.1*):*

The reduction of nitro derivatives to anilines was carried out according to the standard procedure. The compounds **4** and **5** (0.3 g) were dissolved in a mixture of THF and ethanol (1:1) with heating; Raney nickel was added, followed by dropwise addition of hydrazine hydrate (2–3 mL). The reduction was carried out until complete disappearance of the starting compound, testing by TLC (eluent: chloroform – ethanol, 50:1). The products were crystallized from ethanol.

2-(2-Methyl-6-phenylpyrimidin-4-yl)aniline ***6a***

Light yellow solid; mp = 138-139 °C. Yield: 210 mg (72%). ^{1}H NMR (400 MHz, DMSO-d$_6$) δ, 8.30 (m, 2 H, Ar), 8.19 (s, 1 H, CH, pyrimidine), 7.95 (dd, 1 H, *J* = 1.2, 8.0 Hz, Ar), 7.54 – 7.56 (m, 3 H, Ar), 7.19 (td, 1 H, *J* = 1.2, 7.1, 8.0 Hz, Ar), 7.03 (s, 1 H, NH_2), 6.82 (dd, 1 H, *J* = 0.9, 8.2 Hz, Ar), 6.65 (td, 1 H, *J* = 1.0, 7.0, 8.0 Hz, Ar), 2.74 (s, 3 H, Me). ^{13}C

NMR (101 MHz, DMSO-d_6) δ 166.58, 166.06, 162.98, 149.14, 136.80, 131.49, 130.69, 129.71, 128.78 (2 C), 127.18 (2 C), 116.91, 116.78, 115.58, 109.93, 26.08. HRMS(ESI): calculated for $C_{17}H_{16}N_3$ $[M + H]^+$: 262.1339, found: 262.1340; IR ν = 3454, 3290, 3027, 1609, 1567, 1521, 1494, 1372, 1331, 1310, 1254, 1231, 1153, 1029, 870, 785, 752, 736, 694 632 cm^{-1}. Anal. Calcd for $C_{17}H_{15}N_3$: C, 78.13; H, 5.79; N, 16.08. Found: C, 78.16; H, 5.73; N, 16.09.

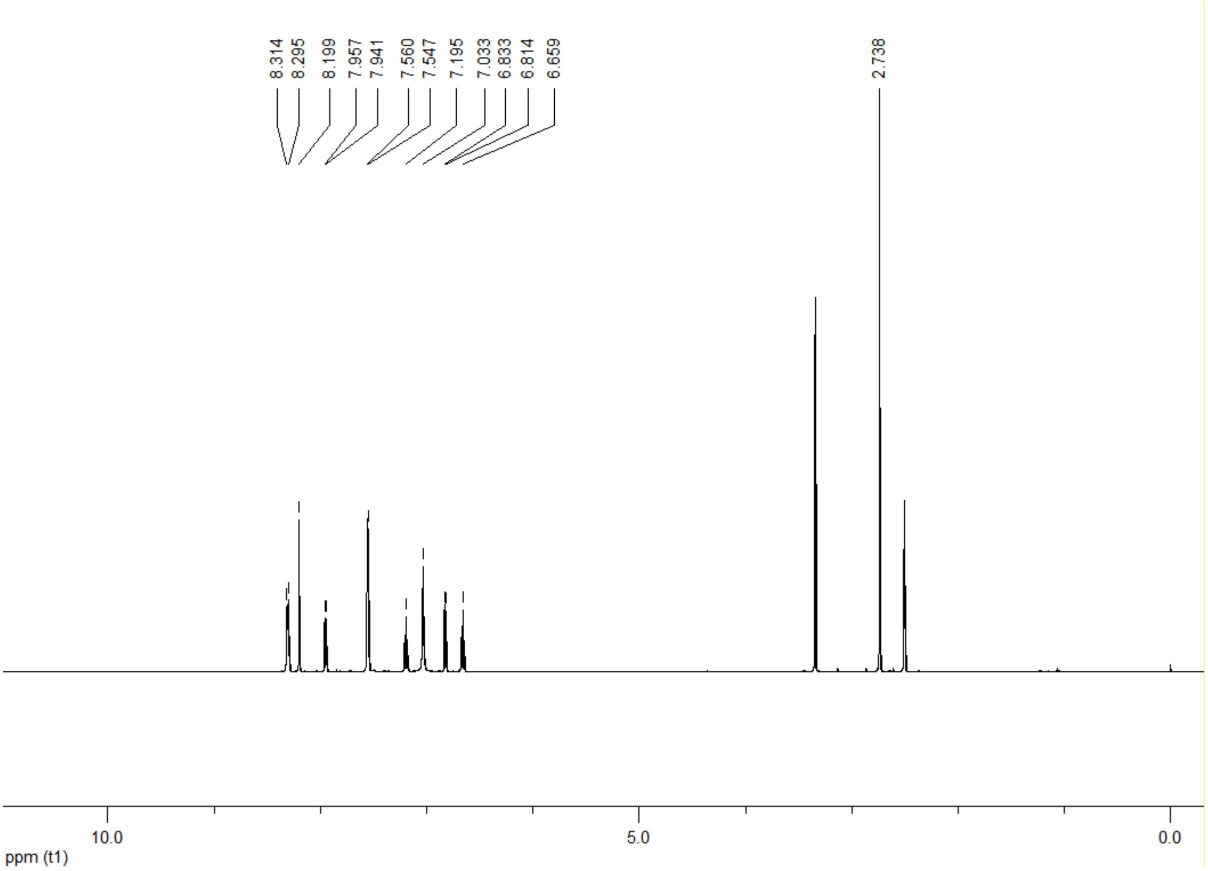

Figure S1. ^1H NMR spectrum of the aniline **6a**.

4-(2-Methyl-6-phenylpyrimidin-4-yl)aniline ***6b***

Yellow solid; mp = 134-135 °C. Yield: 224 mg (86%). ^1H NMR (400 MHz, DMSO-d_6) δ, 8.29 (m, 2 H, Ar), 8.14 (s, 1 H, CH, pyrimidine), 8.09 (m (AA'BB'), 2 H, Ar), 7.52 – 7.56 (m, 3 H, Ar), 6.68 (m (AA'BB'), 2 H, Ar), 5.76 (s, 2 H, NH_2), 2.67 (s, 3 H, Me). ^{13}C NMR (101 MHz, DMSO-d_6) δ 167.01, 164.04, 162.82, 151.80, 136.99, 130.44, 128.67 (2 C), 128.55 (2 C), 126.98 (2 C), 123.17, 113.39 (2 C), 107.12, 26.15. HRMS(ESI): calculated for $C_{17}H_{16}N_3$ $[M + H]^+$: 262.1339, found: 262.1342; IR ν = 3472, 3312, 3194, 3056, 3038, 1633, 1607, 1571, 1527, 1518, 1496, 1453, 1436, 1370, 1306, 1238, 1180, 1075, 832, 761, 691 cm^{-1}. Anal. Calcd for $C_{17}H_{15}N_3$: C, 78.13; H, 5.79; N, 16.08. Found: C, 78.11; H, 5.82; N, 16.05.

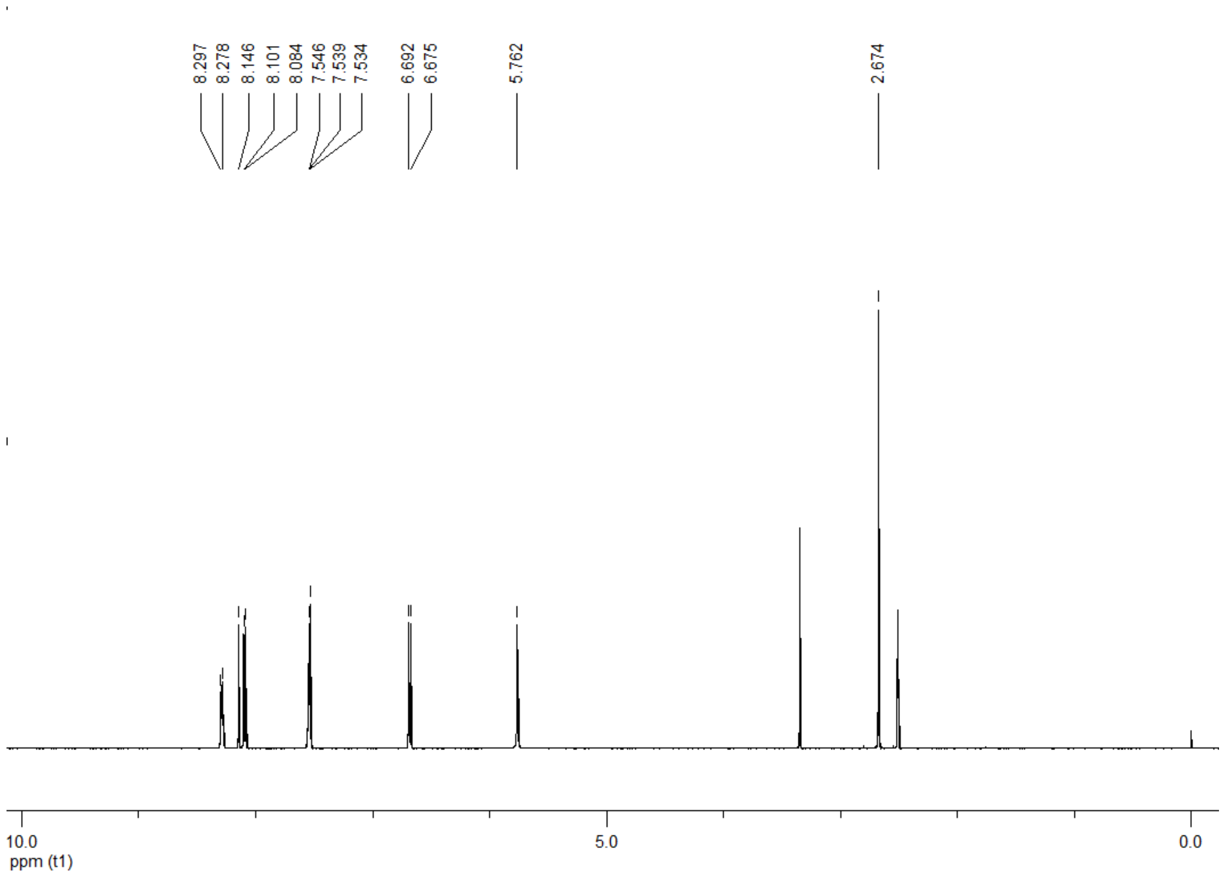

Figure S2. ^{1}H NMR spectrum of the aniline **6b**.

4-(6-(4-Fluorophenyl)-2-methylpyrimidin-4-yl)aniline ***6c***

Light yellow solid; mp = 149-150 °C. Yield: 270 mg (87%). ^{1}H NMR (400 MHz, DMSO-d_6) δ, 8.36 (m (AA′BB′C), 2 H, Ar), 8.15 (s, 1 H, CH, pyrimidine), 8.08 (m (AA′BB′), 2 H, Ar), 7.36 (m (AA′BB′C), 2 H, Ar), 6.67 (m (AA′BB′), 2 H, Ar), 5.76 (s, 2 H, NH_2), 2.66 (s, 3 H, Me). ^{13}C NMR (101 MHz, DMSO-d_6) δ 167.02, 164.66, 164.11, 162.21 (d, 1 C, J = 157.4 Hz), 161.73, 151.88, 133.45 (d, 1 C, J = 3.8 Hz), 129.40 (d, 2 C, J = 11.2 Hz), 128.61 (2 C), 123.08, 115.6 (d, 2 C, J = 27.9 Hz), 113.37 (2 C), 106.94, 26.16. HRMS(ESI): calculated for $C_{17}H_{15}FN_3$ $[M + H]^+$: 280.1245, found: 280.1240; IR ν = 3464, 3316, 3184, 1643, 1600, 1578, 1526, 1506, 1435, 1370, 1307, 1221, 1182, 1161, 1098, 1014, 993, 828, 767 cm^{-1}. Anal. Calcd for $C_{17}H_{14}FN_3$: C, 73.10; H, 5.05; N, 15.04. Found: C, 73.14; H, 5.04; N, 15.05.

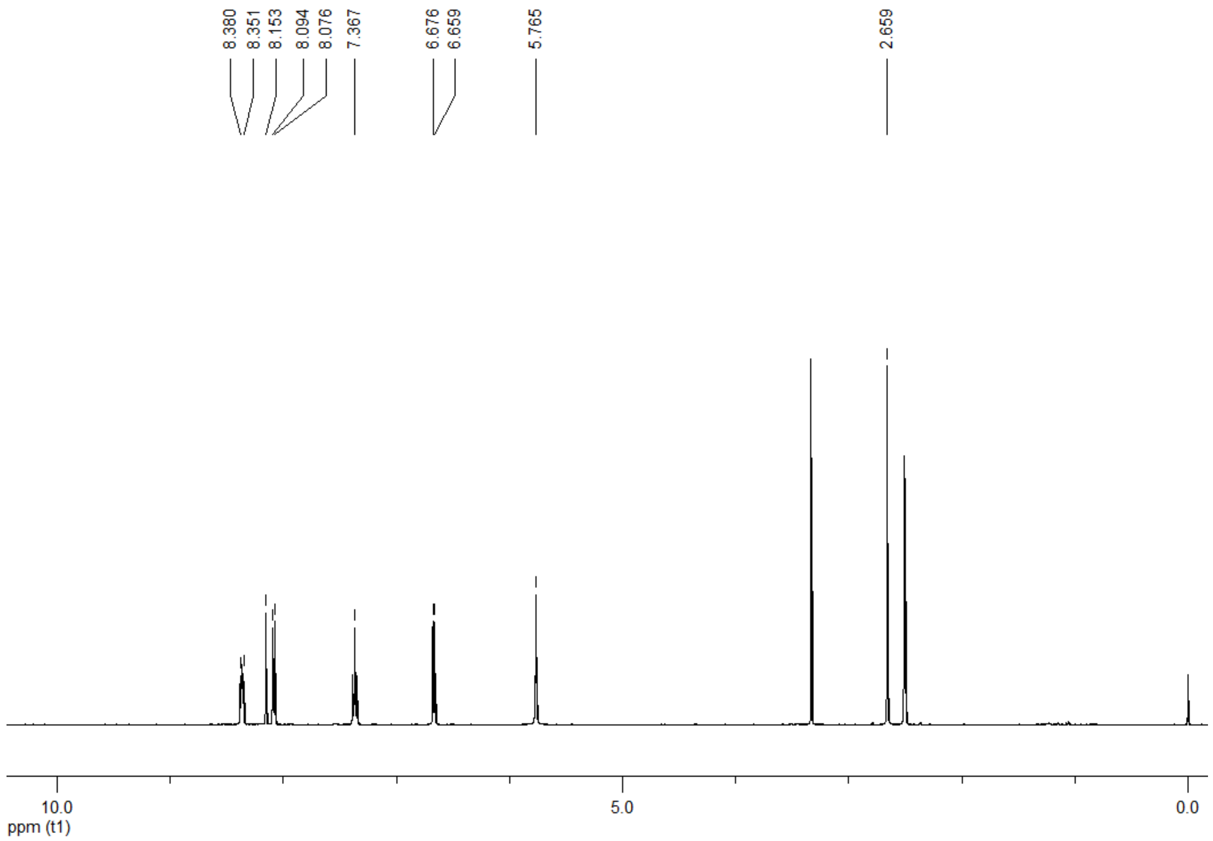

Figure S3. ^{1}H NMR spectrum of the aniline **6c**.

3-(6-(4-Aminophenyl)-2-methylpyrimidin-4-yl)aniline ***6d***

Yellow solid; mp = 171-174 °C. Yield: 225 mg (82%). ^{1}H NMR (400 MHz, DMSO-d_6) δ, 8.02 (m (AA′BB′), 2 H, Ar), 7.95 (s, 1 H, CH, pyrimidine), 7.48 (d, 1 H, *J* = 1.8 Hz, Ar), 7.38 (d, 1 H, *J* = 8.1 Hz, Ar), 7.16 88 (t, 1 H, *J* = 7.8 Hz, Ar), 6.71 (dd, 1 H, *J* = 1.8, 7.8 Hz, Ar), 6.66 (m (AA′BB′), 2 H, Ar), 5.73 (s, 2 H, NH_2), 5.25 (s, 2 H, NH_2), 2.64 (s, 3 H, Me). ^{13}C NMR (101 MHz, DMSO-d_6) δ 166.84, 163.72, 163.65, 151.73, 149.05, 137.60, 129.17, 128.41 (2 C), 123.26, 116.05, 114.65, 113.43 (2 C), 112.17, 106.94, 26.18. HRMS(ESI): calculated for $C_{17}H_{17}N_4$ $[M + H]^+$: 277.1448, found: 277.1448; IR ν = 3451, 3306, 3204, 3053, 3012, 2959, 2920, 2851, 1634, 1607, 1566, 1525, 1514, 1454, 1371, 1311, 1274, 1219, 1186, 1132, 989, 887, 867, 829, 759, 701, 669 cm^{-1}. Anal. Calcd for $C_{17}H_{16}N_4$: C, 73.89; H, 5.84; N, 20.27. Found: C, 73.83; H, 5.85; N, 20.25.

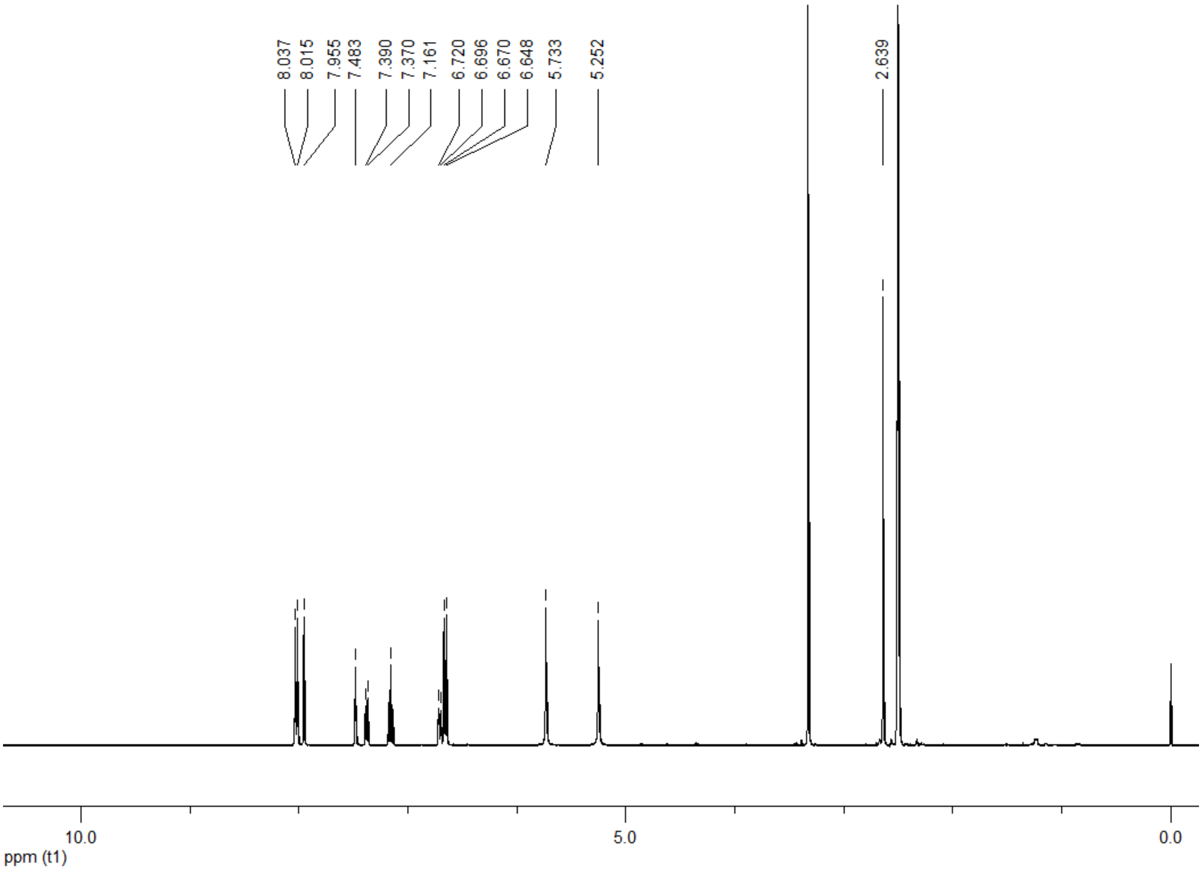

Figure S4. ^{1}H NMR spectrum of the aniline **6d**.

4,4′-(2-Methylpyrimidine-4,6-diyl)dianiline ***6e***

Yellow solid; mp = 200-202 °C. Yield: 215 mg (78%). ^{1}H NMR (400 MHz, DMSO-d_6) δ, 8.01 (m (AA′BB′), 4 H, Ar), 7.89 (s, 1 H, CH, pyrimidine), 6.64 (m (AA′BB′), 4 H, Ar), 5.66 (s, 4 H, NH_2), 2.58 (s, 3 H, Me). ^{13}C NMR (101 MHz, DMSO-d_6) δ 166.53, 163.10 (2 C), 151.43 (2 C), 128.29 (4 C), 123.68 (2 C), 113.38 (4 C), 104.82, 26.24. HRMS(ESI): calculated for $C_{17}H_{17}N_4$ [M + H]$^{+}$: 277.1448, found: 277.1449; IR ν = 3379, 3306, 3181, 3075, 1619, 1574, 1510, 1448, 1430, 1372, 1349, 1293, 1239, 1176, 825, 774, 743, 692 cm^{-1}. Anal. Calcd for $C_{17}H_{16}N_4$: C, 73.89; H, 5.84; N, 20.27. Found: C, 73.85; H, 5.86; N, 20.24.

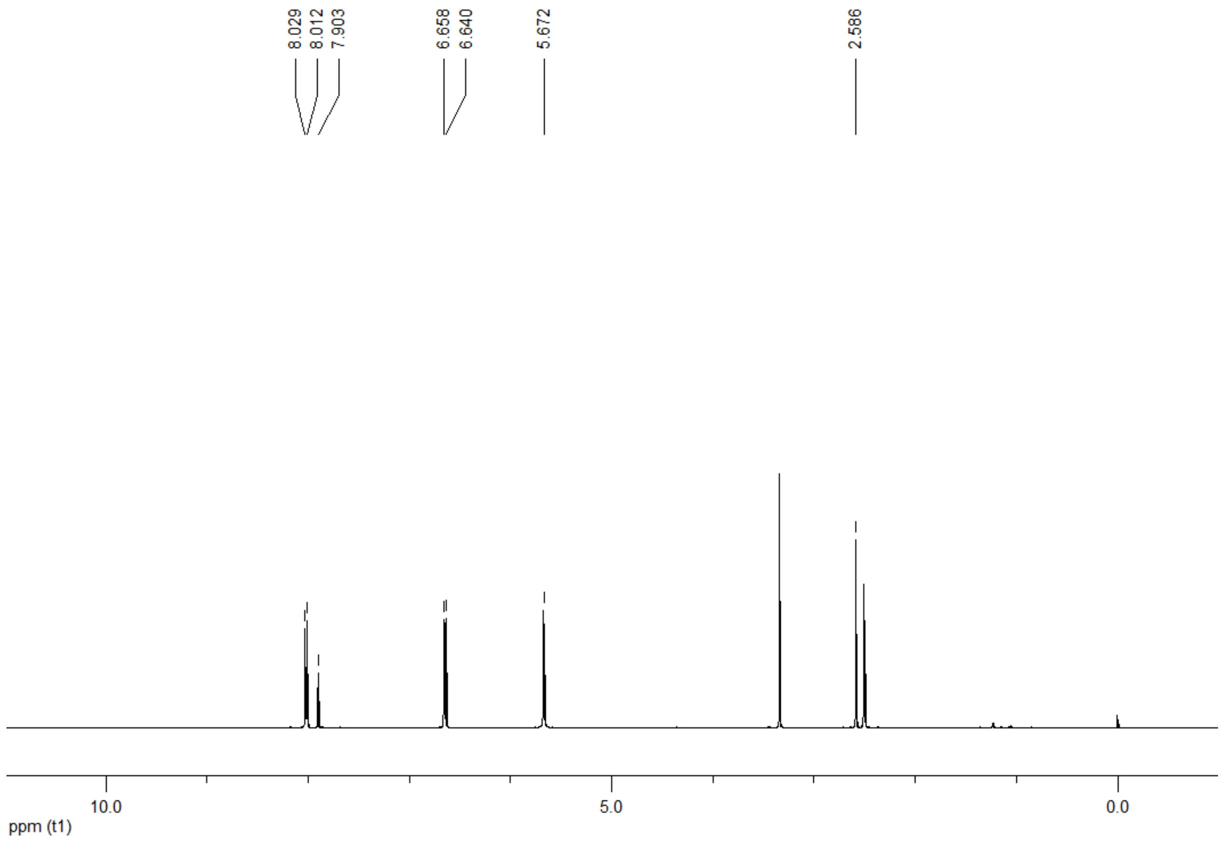

Figure S5. ^{1}H NMR spectrum of the aniline **6e**.

4,4′-(2-Aminopyrimidine-4,6-diyl)dianiline ***6f***

Yellow solid; mp = 255-259 °C. Yield: 200 mg (73%). ^{1}H NMR (400 MHz, DMSO-d_6) δ, 7.90 (m (AA’BB’), 4 H, Ar), 7.32 (s, 1 H, CH, pyrimidine), 6.61 (m (AA’BB’), 4 H, Ar), 6.22 (s, 2 H, NH_2), 5.55 (s, 4 H, NH_2). ^{13}C NMR (101 MHz, DMSO-d_6) δ 164.11 (2 C), 163.57, 150.96 (2 C), 128.01 (4 C), 124.60 (2 C), 113.24 (4 C), 98.22. HRMS(ESI): calculated for $C_{16}H_{16}N_5$ [M + H]$^+$: 278.1400, found: 278.1400; IR ν = 3473, 3440, 3378, 3347, 3168, 1619, 1574, 1564, 1513, 1442, 1427, 1362, 1291, 1241, 1219, 1187, 1176, 815, 796, 785 cm^{-1}. Anal. Calcd for $C_{16}H_{15}N_5$: C, 69.29; H, 5.45; N, 25.25. Found: C, 69.27; H, 5.48; N, 25.22.

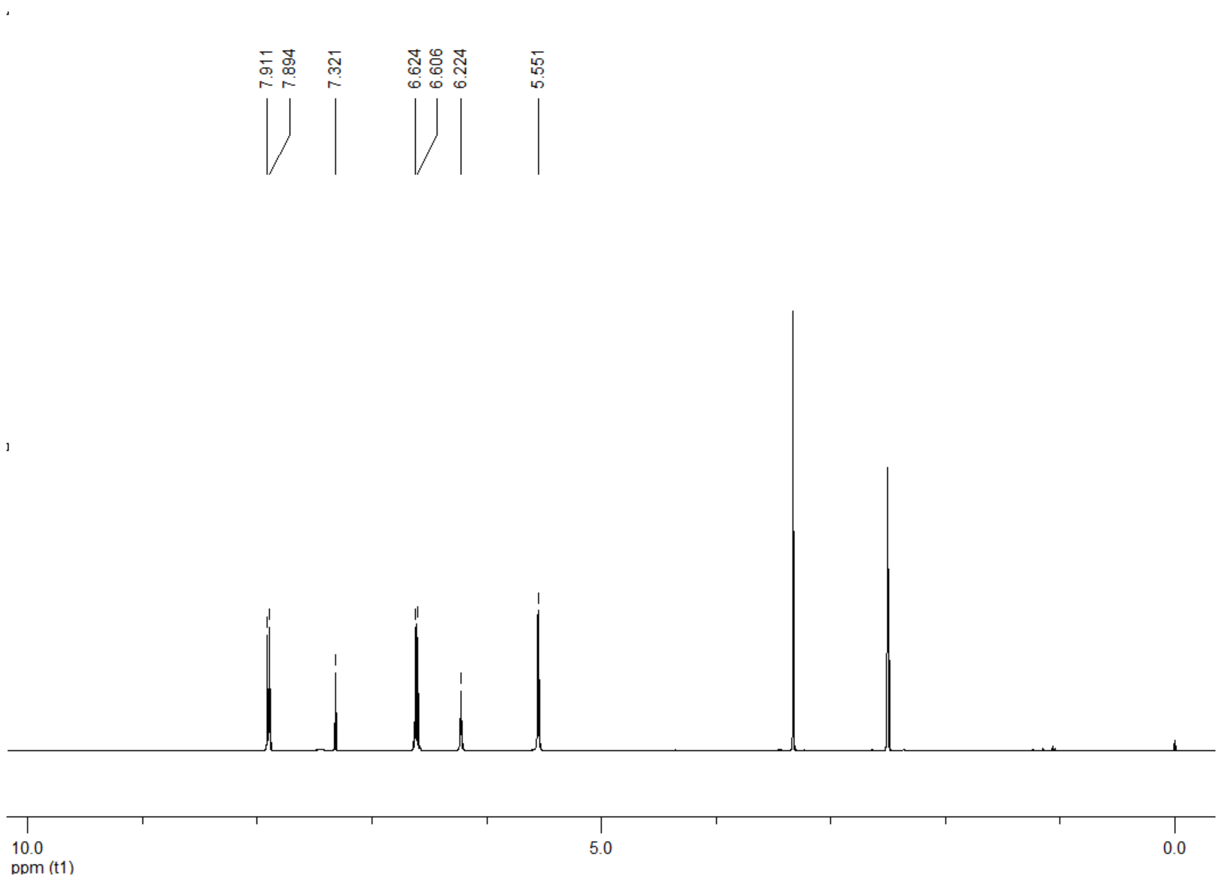

Figure S6. ^{1}H NMR spectrum of the aniline **6f**.

4,4′-(2-(Methylthio)pyrimidine-4,6-diyl)dianiline ***6g***

Yellow solid; mp = 204-205 °C. Yield: 280 mg (87%). ^{1}H NMR (400 MHz, DMSO-d_6) δ, 8.03 (m (AA′BB′), 4 H, Ar), 7.81 (s, 1 H, CH, pyrimidine), 6.66 (m (AA′BB′), 4 H, Ar), 5.75 (s, 2 H, NH_2), 2.59 (s, 3 H, Me). ^{13}C NMR (101 MHz, DMSO-d_6) δ 170.24, 163.19 (2 C), 151.77 (2 C), 128.44 (4 C), 123.04 (2 C), 113.34 (4 C), 103.31, 13.48. HRMS(ESI): calculated for $C_{17}H_{16}N_4SNa$ [M + Na]$^+$: 331.0988, found: 331.0986; IR ν = 3392, 3355, 3303, 3209, 3051, 2919, 1623, 1605, 1564, 1504, 1425, 1402, 1371, 1289, 1236, 1176, 1070, 824, 760 cm^{-1}. Anal. Calcd for $C_{17}H_{16}N_4S$: C, 66.21; H, 5.23; N, 18.17. Found: C, 66.27; H, 5.20; N, 18.21.

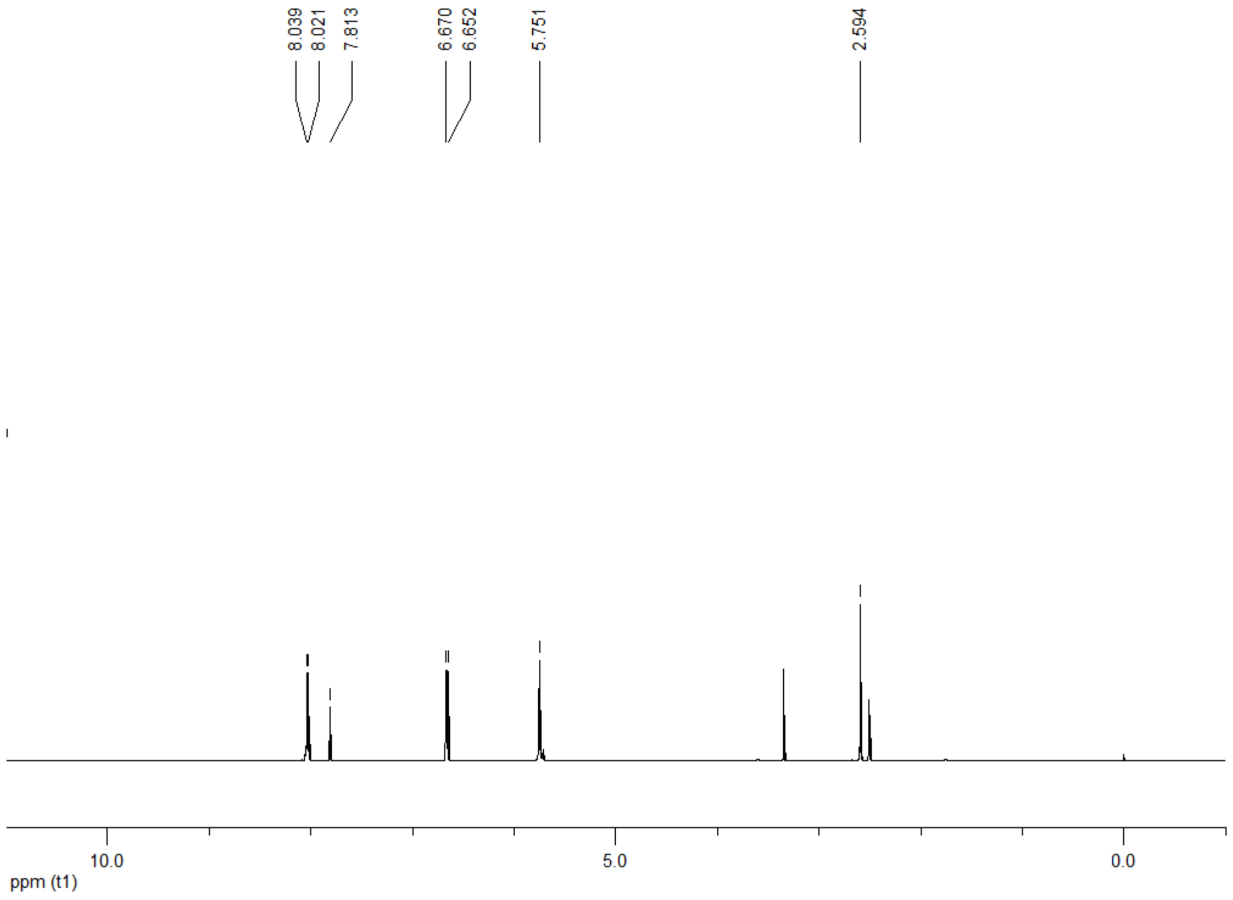

Figure S7. ^{1}H NMR spectrum of the aniline **6g**.

4-(7-(4-Fluorophenyl)-[1,2,4]triazolo[1,5-a]pyrimidin-5-yl)aniline ***7a***

Yellow solid; mp = 239-241 °C. Yield: 255 mg (84%). ^{1}H NMR (400 MHz, DMSO-d_6) δ, 8.56 (s, 1 H, CH, triazolopyrimidine), 8.32 (m (AA'BB'C), 2 H, Ar), 8.13 (m (AA'BB'), 2 H, Ar), 7.63 (s, 1 H, CH, triazolopyrimidine), 7.49 (m (AA'BB'C), 2 H, Ar), 6.69 (m (AA'BB'), 2 H, Ar), 5.93 (s, 2 H, NH_2). ^{13}C NMR (101 MHz, DMSO-d_6) δ 164.65, 162.66, 160.89, 155.67 (d, 1 C, *J* = 208.9 Hz), 152.39, 145.48, 132.32 (d, 2 C, *J* = 23.1 Hz), 129.46 (2 C), 126.35 (d, 1 C, *J* = 8.1 Hz), 122.66, 115.55 (d, 2 C, *J* = 56.8 Hz), 113.47 (2 C), 105.36. HRMS(ESI): calculated for $C_{17}H_{13}FN_5$ [M + H]$^+$: 306.1150, found: 306.1150; IR ν = 3480, 3322, 3208, 3115, 1634, 1597, 1584, 1545, 1502, 1415, 1377, 1304, 1227, 1178, 1163, 917, 824, 775 cm^{-1}. Anal. Calcd for $C_{17}H_{12}FN_5$: C, 66.88; H, 3.96; N, 22.94. Found: C, 66.90; H, 3.95; N, 22.95.

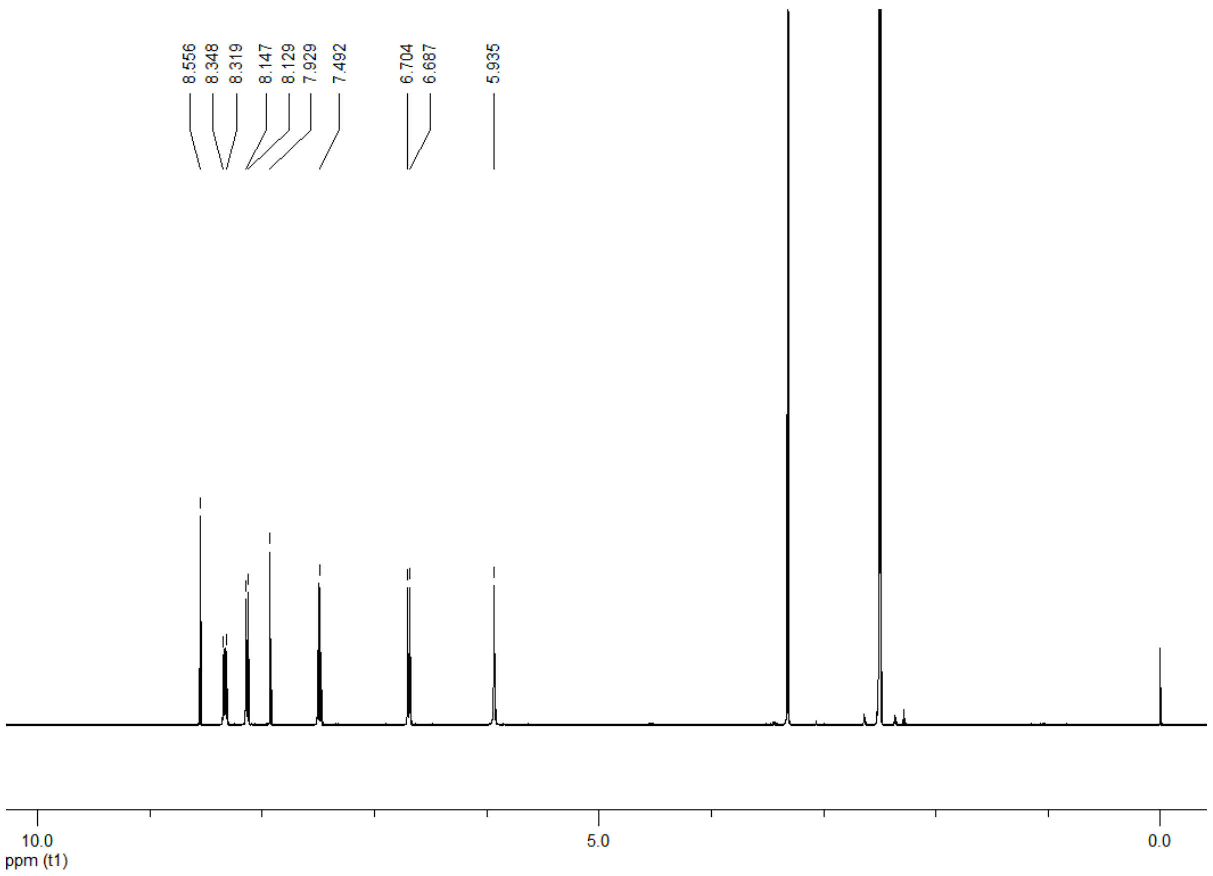

Figure S8. ^{1}H NMR spectrum of the aniline **7a**.

3-(5-(4-Aminophenyl)-[1,2,4]triazolo[1,5-a]pyrimidin-7-yl)aniline ***7b***

Light yellow solid; mp = 267-270 °C. Yield: 250 mg (84%). ^{1}H NMR (400 MHz, DMSO-d$_6$) δ, 8.54 (s, 1 H, CH, triazolopyrimidine), 8.10 (m (AA′BB′), 2 H, Ar), 7.77 (s, 1 H, CH, triazolopyrimidine), 7.38 (t, 1 H, *J* = 1.7 Hz, Ar), 7.28 (dt, 1 H, *J* = 1.4, 7.6 Hz, Ar), 7.25 (t, 1 H, *J* = 7.6 Hz, Ar), 6.82 (dt, 1 H, *J* = 1.7, 7.6 Hz, Ar), 6.69 (m (AA′BB′), 2 H, Ar), 5.92 (s, 2 H, NH$_2$), 5.40 (s, 2 H, NH$_2$). ^{13}C NMR (101 MHz, DMSO-d$_6$) δ 160.76, 156.05, 155.33, 152.31, 148.74, 147.51, 130.76, 129.33 (2 C), 129.06, 122.71, 116.83, 116.61, 114.35, 113.51 (2 C), 104.92. HRMS(ESI): calculated for $C_{17}H_{15}N_6$ [M + H]$^+$: 303.1351, found: 303.1353; IR *ν* = 3460, 3339, 3212, 1628, 1579, 1541, 1513, 1498, 1379, 1280, 1255, 1232, 1217, 1183, 1140, 828, 788, 777, 696 cm^{-1}. Anal. Calcd for $C_{17}H_{14}N_6$: C, 67.54; H, 4.67; N, 27.80. Found: C, 67.57; H, 4.63; N, 27.83.

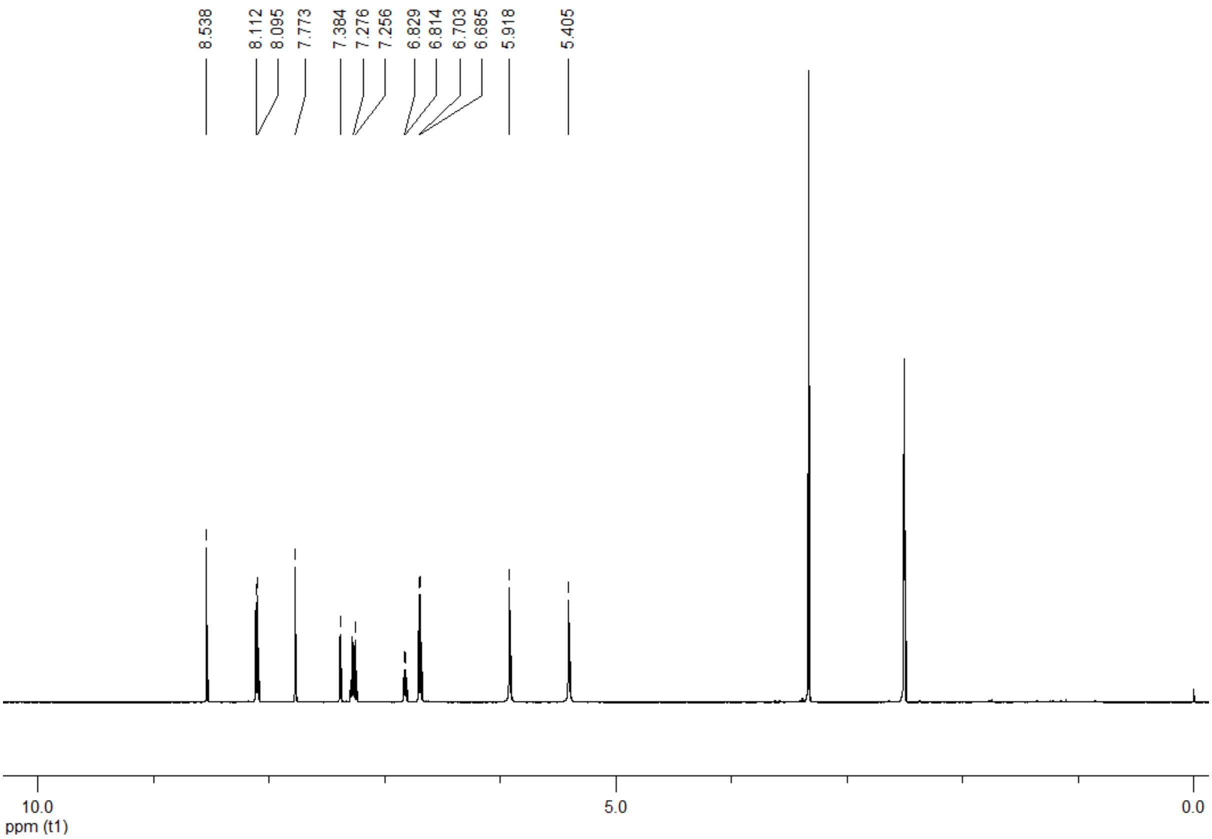

Figure S9. ^{1}H NMR spectrum of the aniline **7b**.

4,4′-([1,2,4]Triazolo[1,5-a]pyrimidine-5,7-diyl)dianiline ***7c***

Yellow solid; mp = 260-263 °C. Yield: 230 mg (77%). ^{1}H NMR (400 MHz, DMSO-d_6) δ, 8.52 (s, 1 H, CH, triazolopyrimidine), 8.19 (m (AA′BB′), 2 H, Ar), 8.11 (m (AA′BB′), 2 H, Ar), 7.77 (s, 1 H, CH, triazolopyrimidine), 6.74 (m (AA′BB′), 2 H, Ar), 6.69 (m (AA′BB′), 2 H, Ar), 6.02 (s, 2 H, NH_2), 5.85 (s, 2 H, NH_2). ^{13}C NMR (101 MHz, DMSO-d_6) δ 160.31, 156.37, 155.08, 152.24, 151.98, 146.97, 131.25 (2 C), 129.18 (2 C), 123.18, 115.94, 113.46 (2 C), 112.94 (2 C), 102.15. HRMS(ESI): calculated for $C_{17}H_{15}N_6$ [M + H]$^+$: 303.1353, found: 303.1358; IR ν = 3467, 3315, 3263, 3110, 3034, 1633, 1589, 1540, 1500, 1380, 1307, 1284, 1240, 1175, 1141, 826, 775 cm^{-1}. Anal. Calcd for $C_{17}H_{14}N_6$: C, 67.54; H, 4.67; N, 27.80. Found: C, 67.55; H, 4.64; N, 27.82.

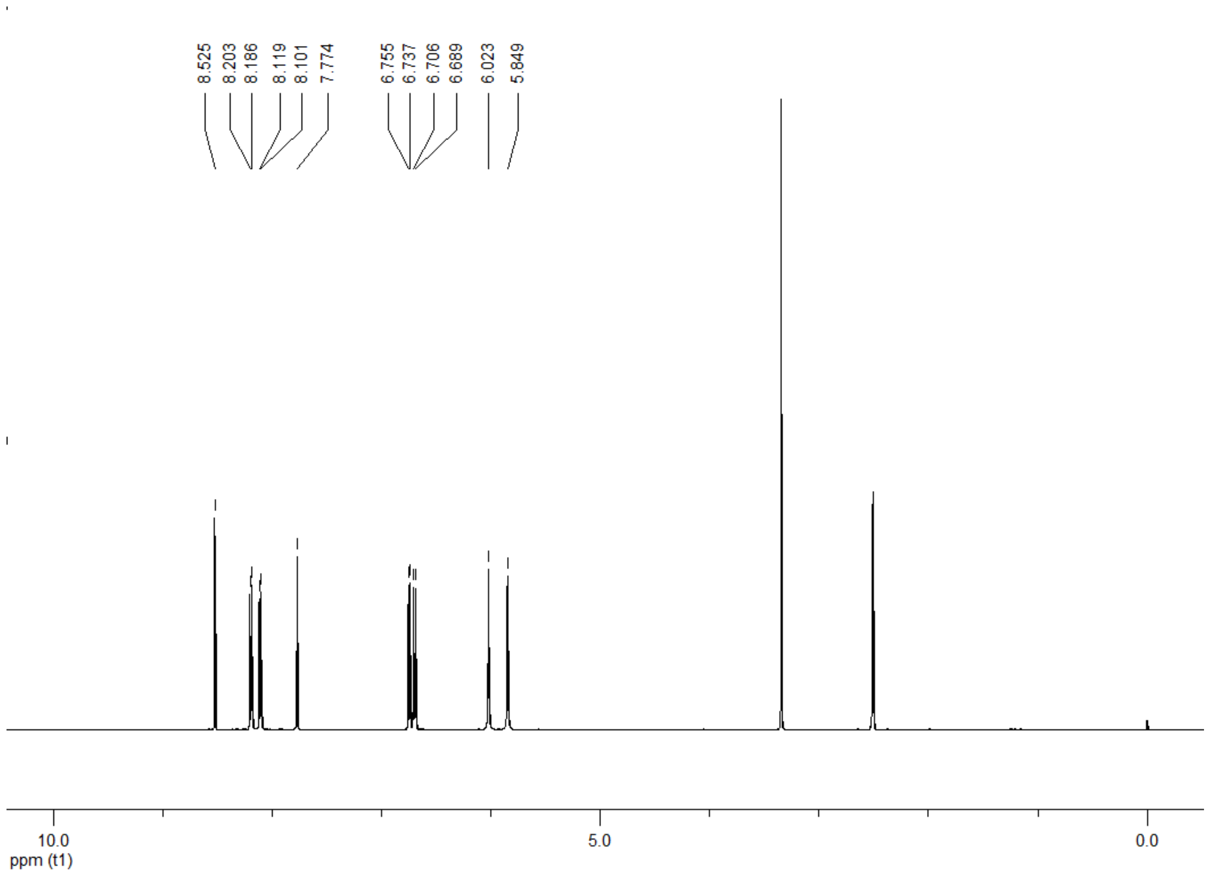

Figure S10. ^{1}H NMR spectrum of the aniline **7c**.

General procedure for the preparation of dihydropyrimidine-5-carboxylate ***10a,b*** (Scheme 2.2; *i, ii*):

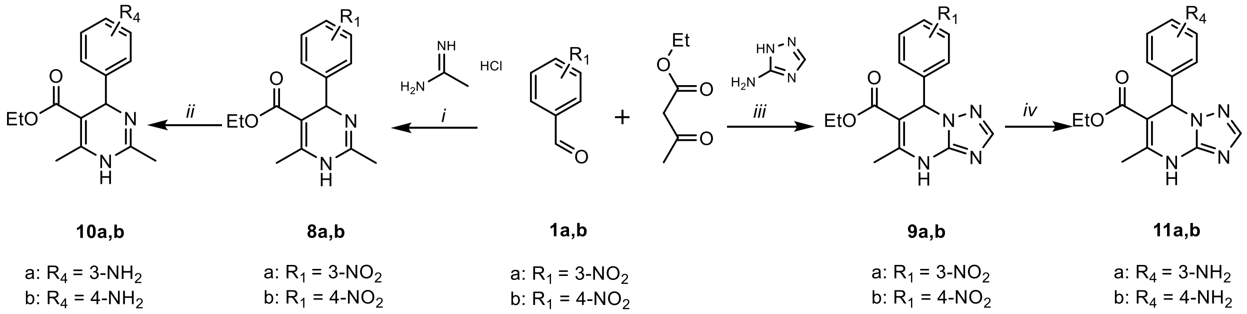

Scheme S2.2

At the first step, nitro-substituted dihydropyrimidines **8a,b** were obtained by a known method [22]. Then the obtained nitrodihydropyrimidine **8a,b** (0.3 g) was dissolved in ethanol and Raney nickel was added, followed by dropwise addition of hydrazine hydrate. The reaction was carried out until the solution color changed from bright yellow to pale yellow. A dispersion was filtered hot. The precipitate that formed after cooling the solution was filtered off.

Ethyl 4-(3-aminophenyl)-2,6-dimethyl-1,4-dihydropyrimidine-5-carboxylate ***10a***

Light yellow solid; mp = 65-67 °C. Yield: 94 mg (35%). ^{1}H NMR (400 MHz, DMSO-d_6) δ 9.53 (br.s, 1H, NH), 6.89 (t, J = 7.7 Hz, 1H, Ar), 6.43-6.36 (m, 3H, Ar), 5.20 (s, 1H, CH), 4.96 (br.s, 2H, NH_2), 4.00-3.94 (m, 2H, CH_2, OEt), 2.20 (s, 3H, Me), 1.88 (s, 3H, Me), 1.10 (t, J = 7.0 Hz, 3H, CH_3, OEt); ^{13}C NMR (101 MHz, DMSO-d_6) δ 166.3, 151.3, 148.4,147.0, 146.2, 128.5, 114.4, 112.5, 112.4, 98.6, 58.8, 56.7, 20.5, 18.6, 14.1; HRMS(ESI): calculated for $C_{15}H_{19}N_3O_2$ [M]$^+$: 273.1477, found: 274.1551; IR ν = 3333, 3210, 3093, 2977, 2929, 1675, 1621, 1604, 1491, 1463, 1370, 1296, 1232, 1168, 1103, 1054, 1018, 865, 782, 700, cm^{-1}.

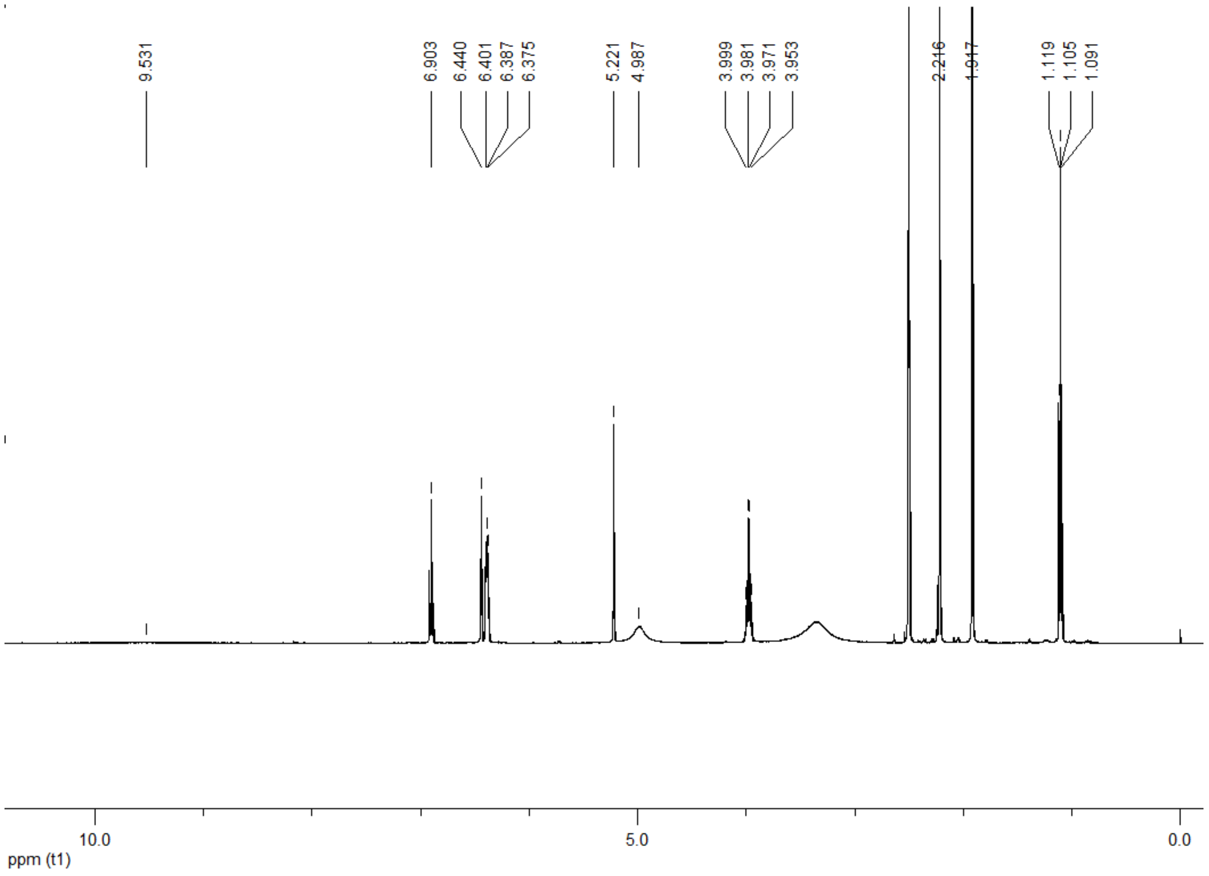

Figure S11. ^{1}H NMR spectrum of the aniline **10a**.

Ethyl 4-(4-aminophenyl)-2,6-dimethyl-1,4-dihydropyrimidine-5-carboxylate ***10b***

Yellow solid; mp = 60-65 °C. Yield: 86 mg (32%). ^{1}H NMR (400 MHz, DMSO-d_6) δ 8.32 (s, 1H, NH), 6.87 (m (AA’BB’), 2H, Ar), 6.46 (m (AA’BB’), 2H, Ar), 5.18 (s, 1H, CH), 4.98 (br.s, 2H, NH_2), 4.00-3.91 (m, 2H, CH_2, OEt), 2.21 (s, 3H, Me), 1.94 (s, 3H, Me), 1.09 (t, J = 6.9 Hz, 3H, CH_3, OEt); ^{13}C NMR (101 MHz, DMSO-d_6) δ 166.0, 152.4, 147.8, 146.1, 132.8, 127.5 (2C), 113.5 (2C), 100.2, 79.1, 59.0, 55.4, 20.0, 18.5, 14.0; HRMS(ESI): calculated for $C_{15}H_{19}N_3O_2$ [M]$^+$: 273.1477, found: 274.1552; IR ν = 3334, 3211, 3098, 2978, 2928, 1675, 1621, 1610, 1513, 1439, 1370, 1232, 1175, 1103, 1054, 1017, 829, 751, 622, 516, cm^{-1}.

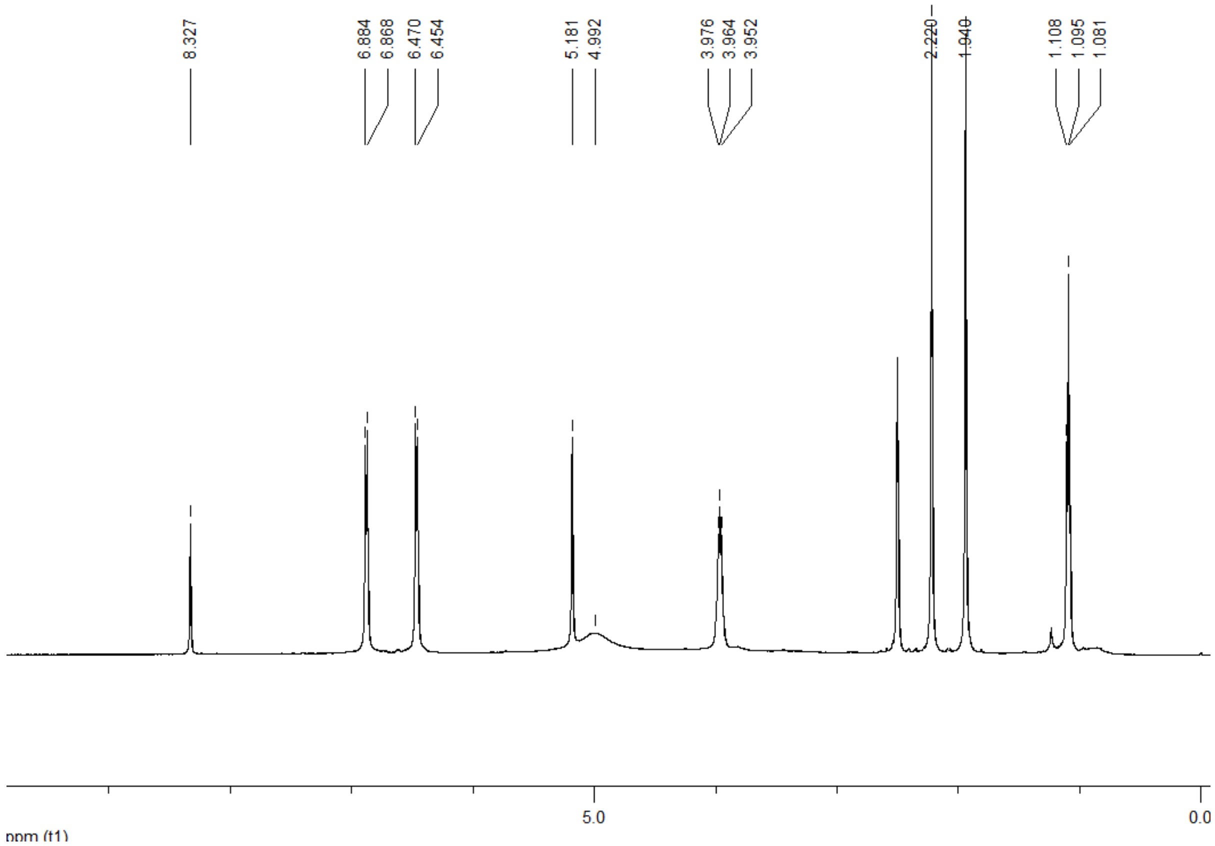

Figure S12. ^{1}H NMR spectrum of the aniline **10b**.

General procedure for the preparation of dihydro-[1,2,4]triazolo[1,5-a]pyrimidine-6-carboxylate ***11a,b*** (Scheme 2.2; *iii*, *iv*):

At the first step, nitro-substituted dihydro-[1,2,4]triazolo[1,5-a]pyrimidine-6-carboxylates **9a,b** were obtained by a known method [23,24]. One-two drops of concentrated HCl were added to a suspension of acetoacetic ester (10 mmol), aminoazole (10 mmol), and the corresponding nitrobenzaldehyde (10 mmol) in ethanol (10 mL). The reaction mixture was refluxed for 5-7 h and then kept at ~20 °C until the reaction was completed (8-10 h). The precipitate that formed was filtered off and purified by recrystallization from ethanol. Then the obtained nitrodihydropyrimidine **9a,b** (0.3 g) was dissolved in ethanol and Raney nickel was added, followed by dropwise addition of hydrazine hydrate. The reaction was carried out until the solution color changed from bright yellow to pale yellow. A dispersion was filtered hot. The precipitate that formed after cooling the solution was filtered off.

Ethyl 7-(3-aminophenyl)-5-methyl-4,7-dihydro-[1,2,4]triazolo[1,5-a]pyrimidine-6-carboxylate ***11a***

Light yellow solid; mp = 233-235 °C. Yield: 168 mg (62%). ^{1}H NMR (400 MHz, DMSO-d_6) δ 10.69 (br.s, 1H, NH), 7.63 (s, 1H, CH), 6.90 (t, J = 7.6 Hz, 1H, Ar), 6.34-6.41 (m, 3H, Ar), 6.08 (s, 1H, CH), 5.07 (s, 2H, NH_2), 4.01-3.93 (m, 2H, CH_2, OEt), 2.39 (s, 3H, Me), 1.08 (t, J = 7.1 Hz, 3H, CH_3, OEt); ^{13}C NMR (101 MHz, DMSO-d_6) δ 165.2, 149.8, 148.6, 146.9, 146.0, 142.5, 128.6, 114.5, 113.4, 113.2, 97.5, 59.5, 59.2, 18.3, 13.9; HRMS(ESI): calculated for $C_{15}H_{17}N_5O_2$ [M]$^+$: 299.1382, found: 299.1382; IR ν = 3432, 3341, 3094, 2980, 2924, 2857, 1688, 1581, 1551, 1465, 1383, 1297, 1244, 1228, 1194, 1167, 1146, 1106, 1173, 1018, 979, 900, 780, 728, 657, 622, 598, 564, cm^{-1}.

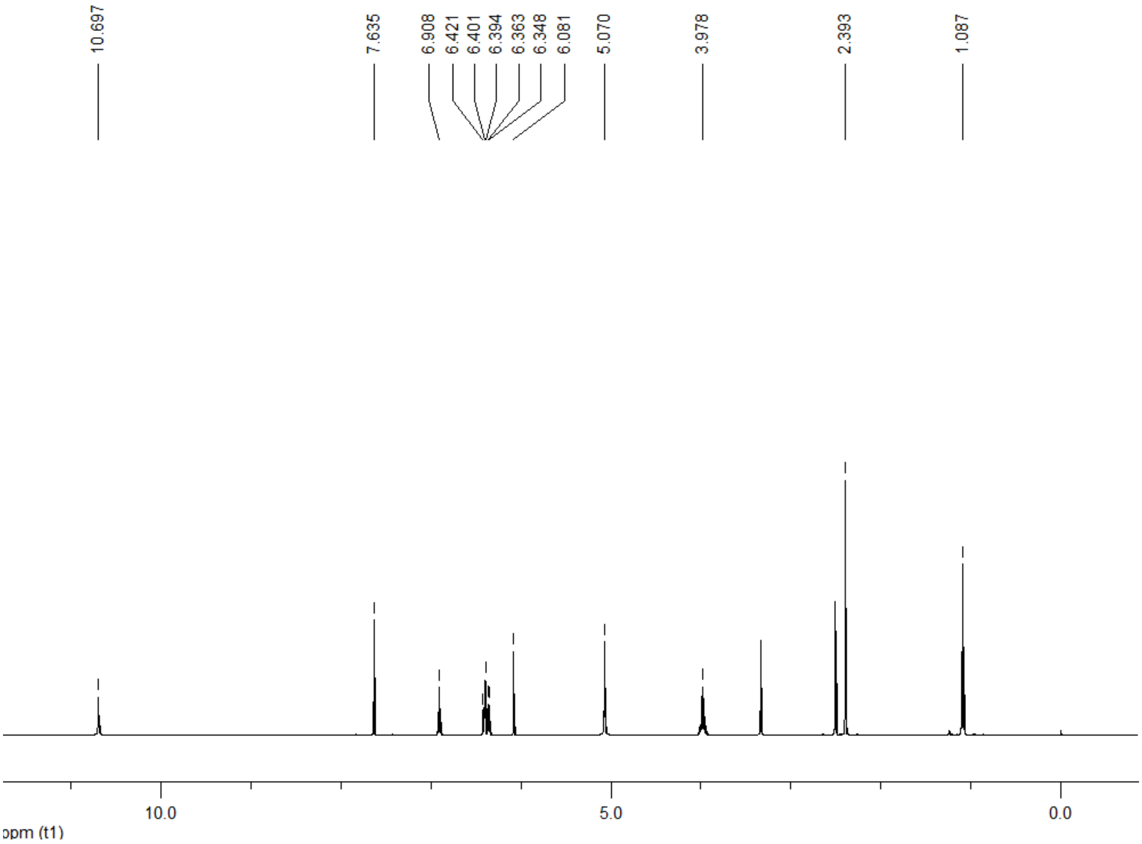

Figure S13. ^{1}H NMR spectrum of the aniline **11a**.

Ethyl 7-(4-aminophenyl)-5-methyl-4,7-dihydro-[1,2,4]triazolo[1,5-a]pyrimidine-6-carboxylate ***11b***

Colorless crystals; mp = 276-127 °C. Yield: 138 mg (51%). ^{1}H NMR (400 MHz, DMSO-d_6) δ 10.62 (br.s, 1H, NH), 7.60 (s, 1H, CH), 6.86-6,82 (m (AA′BB′), 2H, Ar), 6.45-6.42 (m, (AA′BB′), 2H, Ar), 6.07 (s, 1H, CH), 5.05 (s, 2H, NH_2), 4.00-3.92 (m, 2H, CH_2, OEt), 2.38 (s, 3H, Me), 1.07 (t, J = 7.1 Hz, 3H, CH_3, OEt); ^{13}C NMR (101 MHz, DMSO-d_6) δ 165.2, 149.7, 148.3, 146.8, 145.7, 129.3, 127.6, 113.3, 97.8, 59.2, 59.0, 18.2, 13.9; HRMS(ESI): calculated for $C_{15}H_{17}N_5O_2$ [M]$^+$: 299.1382, found: 299.1382; IR ν = 3461, 3338, 3227, 3094,

2984, 2909, 2870, 1694, 1636, 1580, 1556, 1518, 1477, 1317, 1319, 1265, 1248, 1222, 1188, 1147, 1103, 1068, 1018, 981, 898, 825, 806, 774, 731, 698, 658, 579, 528, cm^{-1}.

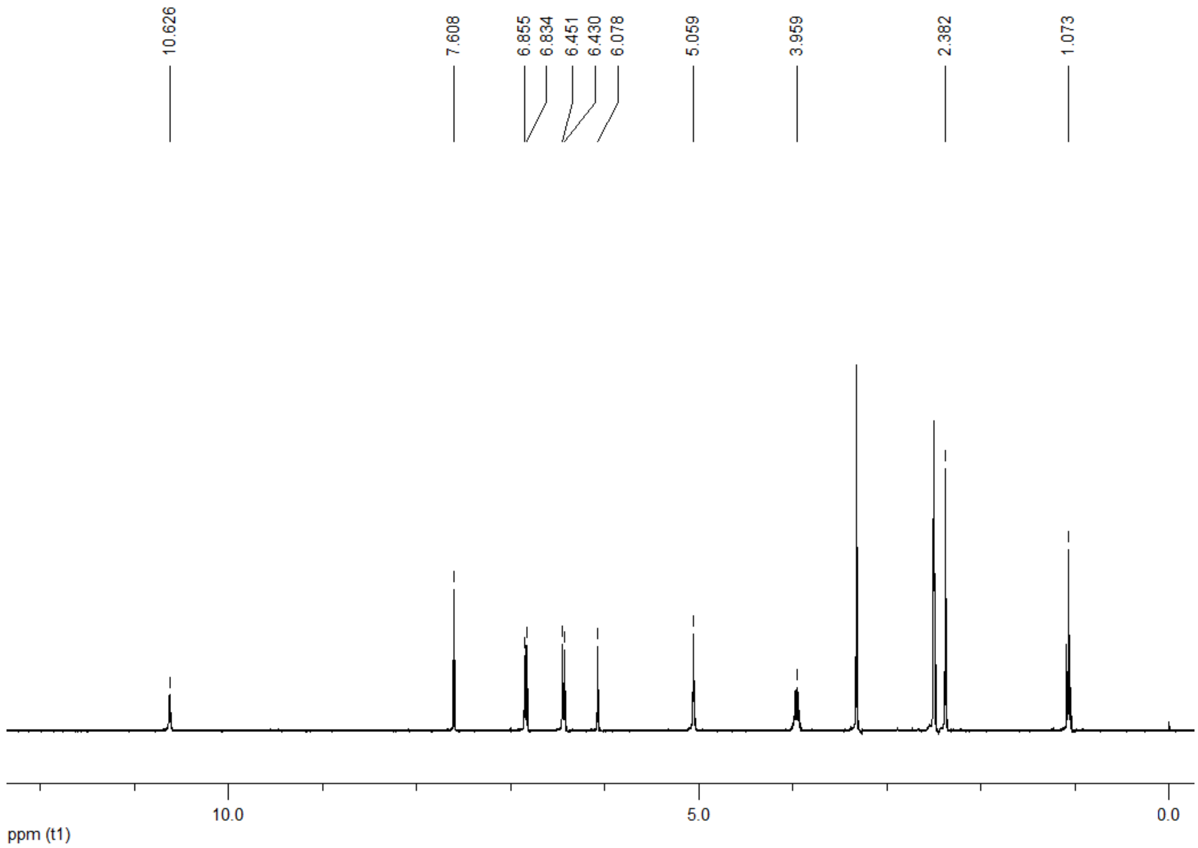

Figure S14. ^{1}H NMR spectrum of the aniline **11b**.
